# Tuning the Biological Activity of Camphorimine Complexes through Metal Selection

**DOI:** 10.3390/antibiotics11081010

**Published:** 2022-07-27

**Authors:** Joana P. Costa, Teresa Pinheiro, Maria S. Martins, M. Fernanda N. N. Carvalho, Joana R. Feliciano, Jorge H. Leitão, Rafaela A. L. Silva, Joana F. Guerreiro, Luís M. C. Alves, Inês Custódio, João Cruz, Fernanda Marques

**Affiliations:** 1 Centro de Química Estrutural, Institute of Molecular Sciences, Departamento de Engenharia Química, Instituto Superior Técnico, Universidade de Lisboa, 1049-001 Lisbon, Portugal; joanavcosta@tecnico.ulisboa.pt; 2 IBB—Instituto de Bioengenharia e Biociências, Departamento de Engenharia e Ciências Nucleares, Instituto Superior Técnico, Universidade de Lisboa, 1049-001 Lisbon, Portugal; teresa.pinheiro@tecnico.ulisboa.pt; 3 C2TN—Centro de Ciências e Tecnologias Nucleares, Instituto Superior Técnico, Departamento de Engenharia e Ciências Nucleares, Instituto Superior Técnico, Universidade de Lisboa, 2695-066 Bobadela, Portugal; mds.martins@campus.fct.unl.pt (M.S.M.); rafaela@ctn.tecnico.ulisboa.pt (R.A.L.S.); joanaguerreiro@ctn.tecnico.ulisboa.pt (J.F.G.); lcalves@ctn.tecnico.ulisboa.pt (L.M.C.A.); i.custodio@campus.fct.unl.pt (I.C.); 4 Departamento de Física, NOVA School of Science and Technology FCT NOVA, Universidade Nova de Lisboa, 2829-516 Caparica, Portugal; jdc@fct.unl.pt; 5 Department of Bioengineering, IBB—Institute for Bioengineering and Biosciences, Associate Laboratory, i4HB—Institute for Health and Bioeconomy at Instituto Superior Técnico, Universidade de Lisboa, Av. Rovisco Pais, 1049-001 Lisbon, Portugal; joana.feliciano@tecnico.ulisboa.pt (J.R.F.); jorgeleitao@tecnico.ulisboa.pt (J.H.L.)

**Keywords:** camphorimine complexes, cytotoxic activity, toxicity, PIXE, ROS

## Abstract

The cytotoxic activity of four sets of camphorimine complexes based on the Cu(I), Cu(II), Ag(I), and Au(I) metal sites were assessed against the cisplatin-sensitive A2780 and OVCAR3 ovarian cancer cells. The results showed that the gold complexes were ca. one order of magnitude more active than the silver complexes, which in turn were ca. one order of magnitude more active than the copper complexes. An important finding was that the cytotoxic activity of the Ag(I) and Au(I) camphorimine complexes was higher than that of cisplatin. Another relevant aspect was that the camphorimine complexes did not interact significantly with DNA, in contrast with cisplatin. The cytotoxic activity of the camphorimine complexes displayed a direct relationship with the cellular uptake by OVCAR3 cells, as ascertained by PIXE (particle-induced X-ray emission). The levels of ROS (reactive oxygen species) formation exhibited an inverse relationship with the reduction potentials for the complexes with the same metal, as assessed by cyclic voltammetry. In order to gain insight into the toxicity of the complexes, their cytotoxicity toward nontumoral cells (HDF and V79 fibroblasts) was evaluated. The in vivo cytotoxicity of complex **5** using the nematode *Caenorhabditis elegans* was also assessed. The silver camphorimine complexes displayed the highest selectivity coefficients (activity vs. toxicity).

## 1. Introduction

Cancer is a leading cause of death worldwide, representing a serious health problem to human societies. Despite significant advances, cancer diagnosis and treatment remain major challenges [1]. Conventional treatment approaches include surgery, radiation therapy, and chemotherapy, used as single treatments or in combination. However, the success of any treatment depends on the type of cancer, its location, and its progression stage [2,3].

Metal-based compounds have been largely used in the therapeutic field due to their unique properties, including their ability to easily undergo redox reactions, variable coordination modes, and reactivity toward organic substrates [4]. Since the discovery of cisplatin, which constituted a milestone in the history of chemotherapy, the search for new complexes with anticancer activity increased hugely [5,6].

Cisplatin has been the most used metal complex to treat a wide spectrum of cancers. Its high efficiency relies on the ability to bind to genomic or mitochondrial DNA and induce lesions in the genetic material, leading to the activation of several signal transduction pathways, which finally leads to the occurrence of necrosis or apoptosis [7]. As a chemotherapeutic drug, its main goal is to kill cancer cells while sparing normal cells and tissues. However, the severe side-effects often associated with the development of resistance that often accompany treatment with cisplatin are still the biggest challenges associated with its use [8]. Some promising alternative, less toxic, and more selective drugs, with a broader spectrum of activity were found, based on metals other than platinum [9,10,11].

Among them, complexes of copper, vanadium, ruthenium, and gold have received considerable interest as anticancer agents due to their promising anticancer properties [12,13,14,15,16]. These complexes, based on a wide variety of ligands, can provide alternatives by presenting different biological profiles and modes of action, with potential to overcome the existing shortcomings associated with the platinum-based drugs [17].

Among gynecological cancers, ovarian cancer is the leading cause of death in women. Unfortunately, in many cases, this type of cancer is diagnosed at an advanced stage, which leads to poor prognosis [18,19,20]. The conventional treatment of ovarian cancer usually involves surgery and chemotherapy, combining a platinum compound and another type of drug with a different mechanism of action to improve efficacy. This procedure succeeds in the early stage of ovarian cancer but fails with patients with advanced disease [21]. Therefore, even though several prevention and treatment options are available, the search for more specific and efficient drugs remains of paramount importance in order to avoid the adverse side-effects caused by the conventional treatments currently available.

In search of alternative metal-based anticancer agents to treat ovarian cancer, Carvalho et al. [22,23] developed silver complexes with camphor-based ligands such as camphor sulfonylimine, camphor carboxylate, and camphor carboxamide. These complexes exhibited higher activity against both cisplatin-sensitive (A2780) and cisplatin-resistant (A2780cisR) ovarian cancer cells compared to cisplatin, while having lower toxicity toward nontumoral cells. Such results suggest that these camphor-derived complexes may be attractive therapeutic alternatives to the pharmacological drugs currently in use.

The aims of the present study were to explore some of these types of ligands (camphorimines) in the synthesis of complexes with different transition metals (copper, silver, and gold), and to evaluate their biological profile as potential alternatives for ovarian cancer treatment.

The cellular uptake of toxicants is a key factor to unveil the cellular targets and mechanisms of action. However, in order to be accurately determined, it is essential to use techniques combining high spatial resolution and high sensitivity for elemental analysis due to the low concentrations of the active species under study. The development of focused ion beam technologies toward a submicrometric scale allowed progress in nuclear microprobes relevant to elemental bioanalysis in single cells [24,25]. Such microprobes make use of high-energy charged particles, usually protons, which enables operating several analytical techniques simultaneously, thereby delivering two-dimensional images of the morphological details and of the micro-distributions of elements in cells. Therefore, it is possible to conclude on the elemental composition and structure of cells with quantitative information [26,27]. Herein, we evaluate the effect of the metal ion on the biological properties of the complexes, particularly the cytotoxic activity, cellular uptake, and cellular distribution, in order to elucidate their mechanisms of action. Furthermore, the toxicity of the most promising complex against the nematode *C. elegans* was also evaluated to gain further insights into the use of these complexes on a living organism.

## 2. Results

**Figure 1:**
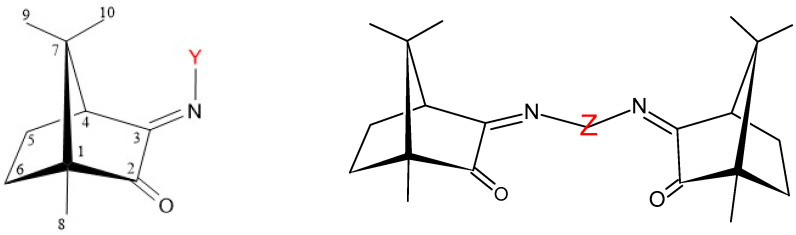
Camphorimine compounds used as ligands: type A (**left**), Y = C_6_H_5_ (^1^A), C_6_H_4_NH_2_ (^2^A); type B (**right**), Z = 4-C_6_H_4_ (^1^B), 3-C_6_H_4_ (^2^B).

Three sets of complexes based on copper, silver, and gold metal sites were synthesized using camphorimine ligands of the mono-(A; OC_6_H_14_NY: Y = C_6_H_5_ (^1^A); C_6_H_4_NH_2_ (^2^A)) and bicamphor (B; (OC_6_H_14_N(μ-C_6_H_4_)NC_6_H_14_O)) types (Figure 1).

The electronic and steric characteristics of the monocamphor complexes were tuned through the imine substituent (Y) at the camphor ligands (^1^A, ^2^A). The new complexes **2**, **4**, and **8** were fully characterized by conventional techniques (NMR, FTIR, and elemental analysis). No structural characterization by X-ray analysis was achieved due to the inability to obtain suitable crystals. However, the structural characterization of related camphorimine complexes showed that coordination to the metal typically involved the nitrogen atom of the imine group [23].

### 2.1. Biological Studies

#### 2.1.1. Cytotoxic Activity

The cytotoxic properties of the camphorimine complexes were evaluated using the MTT assay toward the cisplatin-sensitive ovarian cancer cells A2780 and OVCAR3, as well as the normal HDF and V79 fibroblasts. The overall observation was that the copper complexes exhibited lower anticancer activity than the silver and gold complexes (Table 1), regardless of the oxidation state of the copper site Cu(I) (**1**, **3**) or Cu(II) (**2**, **4**), or the mono- (**1**, **2**, **4**) or bicamphor (**3**) characteristic of the camphorimine ligands. Apparently, the Cu(II) complexes (**2**, **4**) displayed lower cytotoxicity than Cu(I) complexes (**1**, **3**) toward normal cells (V79 and HDF).

The gold complexes (**8**, **9**) displayed IC_50_ values ca. two orders of magnitude lower than the copper complexes (Table 1) for cancer cells (A2780 and OVCAR3) or normal cells (HDF and V79 fibroblasts), with a slightly higher selectivity (SI). These results indicate a better anticancer performance of the gold compared to the copper complexes. However, among the camphorimine complexes under study (Cu, Ag, and Au), the silver complexes displayed the most promising results. Although their IC_50_ values toward cancer cells were higher than those of the gold complexes, they exhibited a very low toxicity toward normal cells and a high selectivity (SI) toward A2780 and OVCAR3 cancer cells (Table 1), which is a highly relevant aspect concerning biological applications.

A closer look into the cytotoxic activity vs. the characteristics of the silver complexes suggested that the lipophilicity of the ligand and the geometry of the complex were non-innocent parameters concerning their cytotoxic activity. Although further results are necessary to support the observation, according to the lipophilicity data (partition coefficient in octanol/water predicted using ACDC software) for complexes **5** (^1^A, 2.94 ± 0.58) and **7** (^2^A, 2.25 ± 0.59), an increase in the lipophilicity enhanced the anticancer selectivity. A direct relationship between the antimicrobial activity of the silver camphorimine complexes and their lipophilicity was previously reported [28].

The camphorimine ligands by themselves displayed no cytotoxic activity, as assessed under similar experimental conditions (>100 μM, data not shown).

The IC_50_ values measured for each complex (Table 1) demonstrate that they exhibited similar cytotoxicity toward the two ovarian cancer cell lines. Therefore, the subsequent studies were performed with the OVCAR3 cells.

For comparative purposes, the activity of salt precursors CuCl and CuCl_2_, AgNO_3_ and Ag(CH_3_COO), and KAu(CN)_2_, was evaluated using the OVCAR3 cells, and the respective IC_50_ values were calculated (Table 1). With the exception of complexes **4** (Cu(II)) and **7** (Ag(I)), all the other complexes had lower IC_50_ values than the corresponding metal precursors.

The cytotoxic activity of the reference drug cisplatin was evaluated in both ovarian cancer cells, using the same experimental conditions. The IC_50_ values found at 24 h incubation were higher than those found for the silver and gold complexes, 21 ± 5.0 μM and 13 ± 4.7 μM for the A2780 and the OVCAR3 cells, respectively.

The cytotoxicity against the noncancer cell lines was determined to calculate the selectivity index (SI value), a parameter used to express a compound’s in vitro efficacy. The cytotoxicity screening was conducted using two normal fibroblasts cell lines to obtain the selectivity index. As can be observed from Table 1, the IC_50_ values obtained for the two cell lines differed, particularly for copper complex **1** and silver complexes **6** and **7**. Therefore, the HDF human cell line was selected for the calculation of the SI values.

Results were analyzed on the basis of the assumption that an SI value <2 indicates general toxicity of a compound, while an SI value ≥10 indicates that the compound has potential to be further investigated as a therapeutic drug [29]. The IC_50_ values obtained for HDF were compared to those obtained for OVCAR3 (Table 1). We found that the majority of the compounds had SI values higher than 2, with the copper complexes being less selective. The silver complexes were more selective, particularly complex **5**, that exhibited a high degree of cytotoxic selectivity (SI > 50).

#### 2.1.2. Complex Stability in Solution

**Figure 2:**
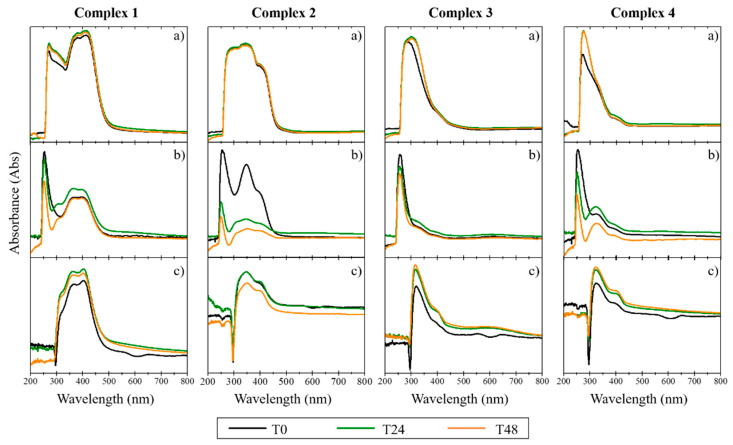
UV/Vis spectra of copper complexes (**1**, **2**, **3** and **4**) in DMSO solution (**a**), and in phenol red-free DMEM in the absence (**b**) and presence of FBS (**c**). The data were collected at T = 0 h (T0), T = 24 h (T24), and T = 48 h (T48) after creating the complex solutions.

**Figure 3:**
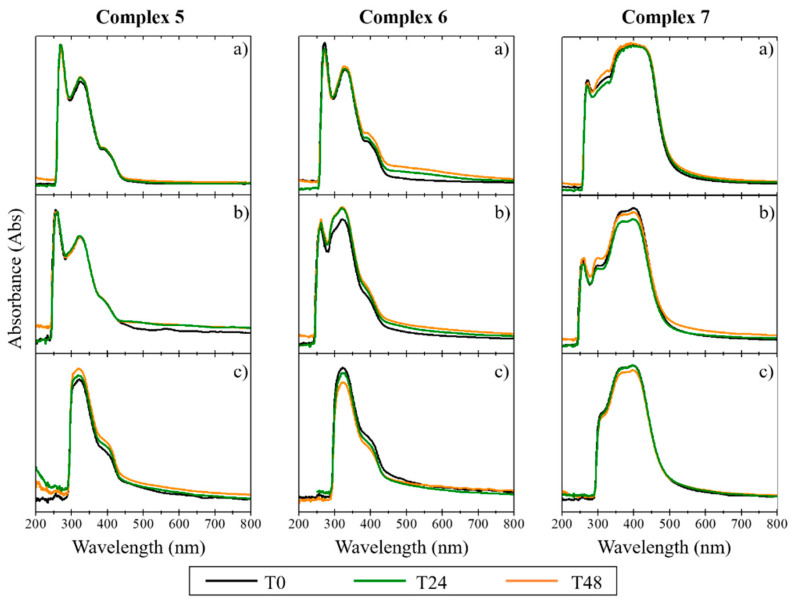
UV/Vis spectra of silver complexes (**5**, **6**, and **7**) in DMSO solution (**a**), and in phenol red-free DMEM in the absence (**b**) and presence of FBS (**c**). The data were collected at T = 0 h (T0), T = 24 h (T24), and T = 48 h (T48) after creating the complex solutions.

**Figure 4:**
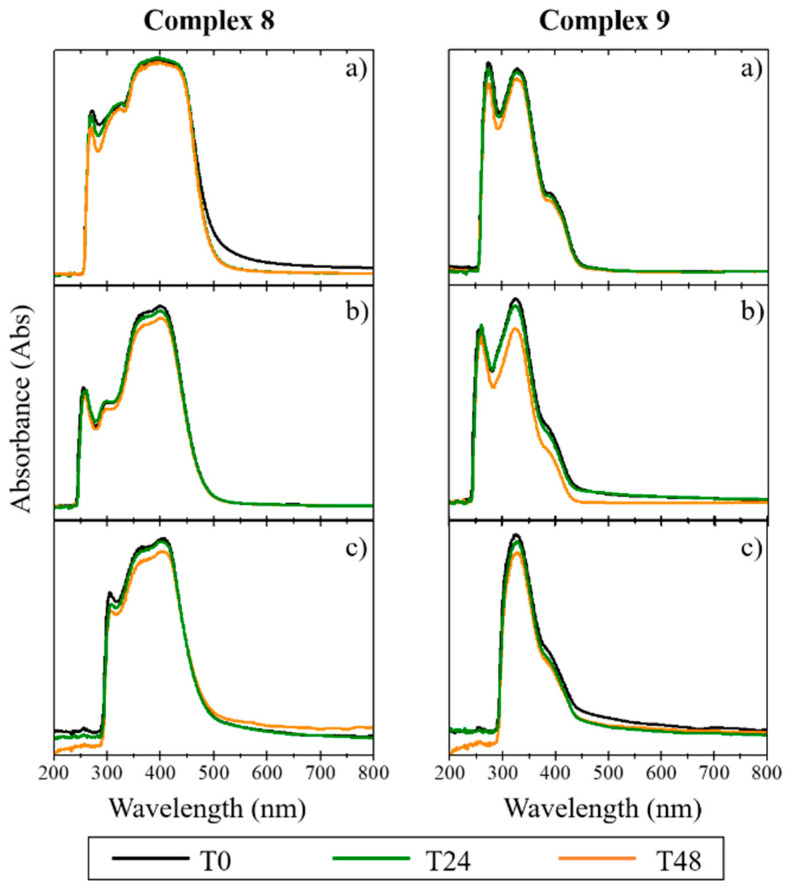
UV/Vis spectra of gold complexes (**8** and **9**) in DMSO solution (**a**), and in phenol red-free DMEM in the absence (**b**) and presence of FBS (**c**). The data were collected at T = 0 h (T0), T = 24 h (T24), and T = 48 h (T48) after creating the complex solutions.

Since biological studies are carried out in aqueous media at physiological pH, it is necessary to ensure that the complexes do not precipitate in the aqueous milieu and that they are stable on the timescale of the studies. The stability in cellular medium of the complexes was evaluated for 48 h, using UV/Vis spectroscopy [30]. The absorption spectrum was recorded for each complex dissolved in (a) 100% DMSO, (b) colorless DMEM with 1% DMSO, and (c) colorless DMEM with 1% DMSO + 10% FBS [30]. For the copper complexes, the results demonstrated a depletion of intensity in the characteristic peaks of the spectrum with the addition of DMEM and FBS. The disappearance of the initial absorption band between 200 and 300 nm for the copper complexes (Figure 2) suggests the occurrence of some degradation during the study (48 h), slightly lower in the case of complex **1**. The Ag(I) and Au(I) complexes were found to be more stable than the copper complexes, as confirmed by the maintenance of their absorption bands between 300 and 400 nm (in the three different compositions and with temporal evolution; Figure 3 and Figure 4). The results indicate that these complexes maintained their original form in solution, even after 48 h incubation time. As low concentrations were used in the biological studies, we expect that both the stability and the solubility were not impaired.

#### 2.1.3. Complex–DNA Interaction

**Figure 5:**
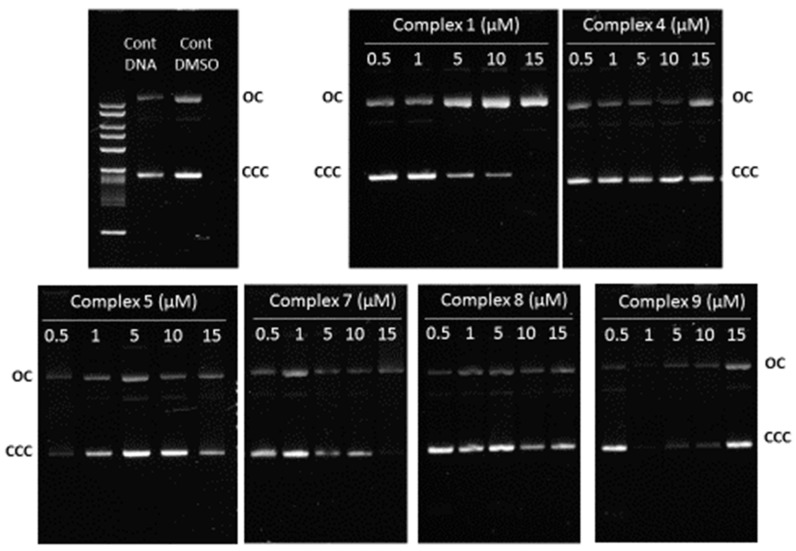
Assessment of the interaction of the complexes with DNA based on the evaluation of the samples’ electrophoretic mobility. Supercoiled ϕX174 DNA was incubated with 0.5, 1, 5, 10, and 15 µM of complexes **1**, **4**, **5**, **7**, **8**, and **9** for 24 h at 37 °C, before samples were resolved using agarose gel electrophoresis. Open circular (OC) and covalently closed circular (CCC) forms of DNA are identified. The results for the respective ϕX174 DNA controls incubated with and without DMSO are also shown.

The obtained results show that the copper complexes were less cytotoxic and less stable in culture medium. Therefore, we focused our studies mostly on the silver (**5** and **7**) and gold complexes (**8** and **9**) having the monocamphor ligands ^1^A and ^2^A, respectively. Complex **1** was also included for comparison. We evaluated the complexes’ ability to interact with DNA, by visualizing in vitro conformational changes in ΦX174 supercoiled DNA using agarose gel electrophoresis assays [30]. As shown in Figure 5, none of the complexes led to changes in the electrophoretic mobility of ϕX174 DNA. Consequently, it is possible to conclude that none of them interacted significantly with DNA molecules. These results strongly suggest that their cytotoxicity was mediated by a mechanism of action distinct from that of cisplatin, which was previously shown to bind to DNA and induce extensive electrophoretic mobility changes in DNA [30].

#### 2.1.4. Production of ROS

ROS are highly reactive and unstable molecular species, leading to oxidative stress and triggering the activation of cellular death mechanisms. The main species include superoxide (O_2_^•−^), hydrogen peroxide (H_2_O_2_), peroxynitrite (ONOO^−^), and hydroxyl radical (OH^•^) [31]. The preferred molecular targets are mainly proteins, DNA and mitochondrial DNA, and lipids. In particular, ROS can disrupt the lipid membrane and increase membrane fluidity and permeability [32]. A great number of new drugs are being developed to elevate ROS levels inducing oxidative stress incompatible with cell viability [33].

The molecular probe dichlorodihydrofluorescein diacetate (H_2_DCF-DA) was used to assess the levels of ROS, e.g., hydrogen peroxide, hydroxyl radicals, and peroxynitrite, generated in response to the effect of the complexes. This compound suffers oxidation and produces a fluorescent compound (DCF) in the presence of ROS [31]. The induction of intracellular ROS by the compounds (metal precursors and copper, silver, and gold complexes) was analyzed at λ_exc._ = 492 nm 517 nm emission using a Varioskan LUX scanning multimode reader (Thermo Fisher Scientific).

**Figure 6:**
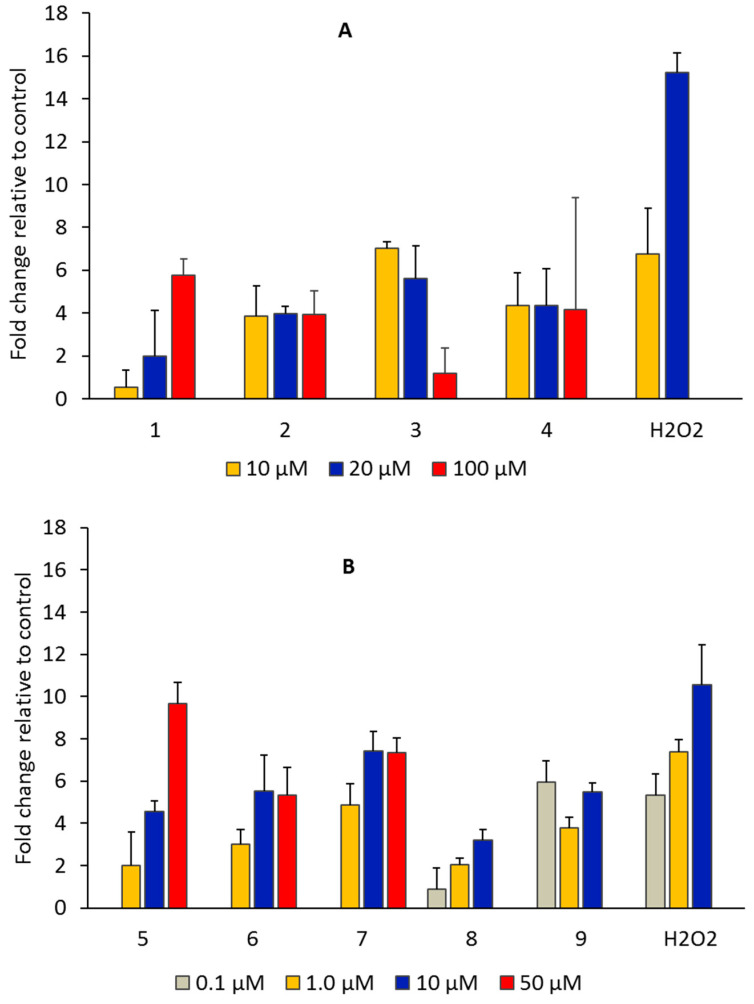
Production of ROS (H_2_O_2_, ONOO^−^, and OH^•^) in OVCAR3 cells, as probed with H_2_DCF-DA. Cells were treated with complexes (**A**) 1, 2, 3, and 4 at 10, 20, 50, and 100 µM, and complexes (**B**) 5, 6, 7, 8, and 9 at 0.1, 1.0, 10, 20, and 50 µM, using the H_2_DCFDA method based on the detection of DCF fluorescence. For comparative purposes, H_2_O_2_ was included in each assay as a positive control.

As depicted in Figure 6, all the complexes induced the production of ROS (relative to untreated cells) in OVCAR3 cells. The metal precursors were much less effective in inducing ROS generation when incubated with OVCAR3 cells, according to the fluorescence intensity (fold change relative to control well below 1, Supplementary Figure S1). A correlation among the cytotoxic activity, the levels of ROS, and the redox potentials (Table 1) was apparent, since the Au(I) complexes displayed the lower reduction potentials (Table 1), the higher levels of ROS, and the higher cytotoxic activities (Figure 6, Table 1). The Ag(I) complexes that were reduced at intermediate values between gold and copper complexes displayed intermediate IC_50_ values (Table 1) and levels of ROS (Figure 6).

**Figure 7:**
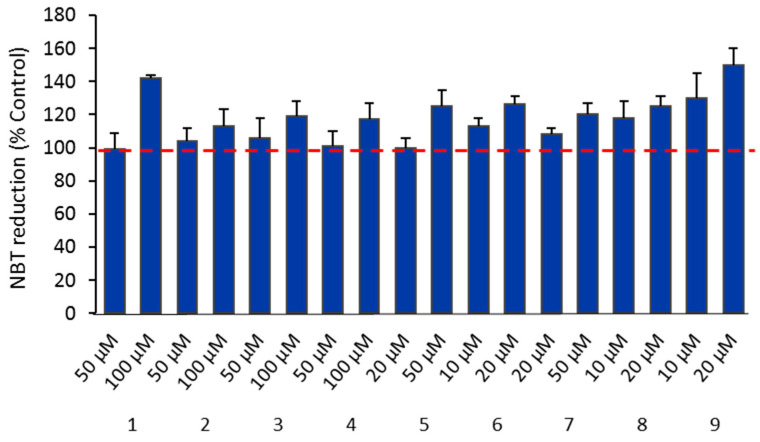
Dose-dependent increase in NBT reduction after treatment of OVCAR3 cells with the camphorimine complexes. Cells were treated with complexes **1**, **2**, **3**, and **4** at 50 and 100 µM, complexes **5** and **7** at 20 and 50 µM, and complexes **6**, **8**, and **9** at 10 and 20 µM.

The ROS species superoxide (O_2_^•−^) can be detected with NBT, a cell-permeant dye that can cross the cell membrane and be reduced by intracellular free radicals (superoxide anions) to form formazan particles, which can be detected as blue precipitates in the cells. As shown in Figure 7, complexes **1**, **5**, and **9** increased the ROS levels in a dose-dependent manner, with ROS production being considerably higher when compared with controls (untreated cells).

#### 2.1.5. Membrane Lipid Peroxidation

Lipid peroxidation is a process in which ROS react with C=C double bonds in lipids, leading to a mixture of products such as hydroperoxides and malondialdehyde (MDA), one of the final products of polyunsaturated fatty-acid peroxidation in the cells. The MDA level is commonly known as a biomarker for lipid peroxidation [34].

Measurement of MDA relies on the detection of thiobarbituric acid (TBA)-reactive compounds generated from the decomposition of lipid peroxidation products. This reaction, which takes place under acidic conditions at 95 °C, results in the formation of an MDA–TBA adduct that can be quantified by its absorbance at 532 nm. Results in Table 2 show that the content of MDA in OVCAR3 cells treated with **1**, **5**, and **9** was low but higher than the basal level of lipid peroxides in the untreated cells (7 ± 4 pmol/10^6^ cells). Additionally, the MDA levels induced by the complexes correlated with the levels of ROS, pointing the involvement of ROS production in the mechanism of action of complexes under study and, consequently, the peroxidation of the membrane lipids, as supported by the correlation between the production of ROS and the levels of lipid peroxides, particularly for complexes **5** and **9**.

One of the ROS species conceivably responsible for MDA formation is superoxide (O_2_^•−^) formed through electron transfer to the camphorimine complexes. In processes involving metal sites, superoxide production is enhanced at potentials close to 0 V [35]. Among the camphorimine complexes under study, the silver complexes were those that better fit the potential requirements (Table 1) to enable superoxide formation and lipid peroxidation on cancer cells. The highest levels of lipid peroxides formed in the presence of camphorimine complexes were registered for cells treated with the silver complex **5** (Table 2), which was reduced at a potential close to 0 V ($E_{p}^{red}$ = 0.12 V). Reduction of Au(I)→Au(0) at **9** and Cu(I)→Cu(0) at **1** occurred at much lower potentials (Table 1). Different levels of lipid peroxidation in cancer and healthy cells conceivably drove the high selectivity (SI) found for the silver camphorimine complexes (Table 1).

#### 2.1.6. Caspase-3/7 Activation

**Figure 8:**
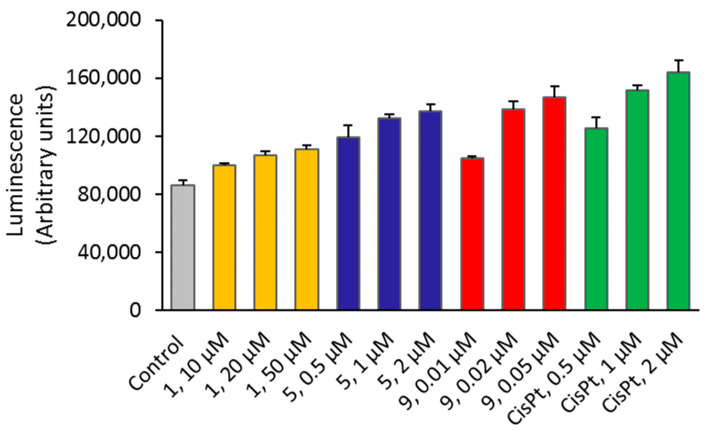
Caspase activity assays. Apoptosis was evaluated by CaspaseGlo assay after cell treatment for 24 h, using for each complex at concentrations below their IC_50_ value. Data was expressed as the mean ± SD, in arbitrary luminescent units. Cisplatin was included for comparison. **1** (yellow), **5** (blue), **9** (red), and cisplatin (green).

Caspases are a family of cysteine proteases playing central roles in the regulation of cellular processes such as programmed cell death. Among them, caspase-3 and -7 are considered executioners of the apoptosis pathways. We investigated the possible activation of apoptotic events in OVCAR3 cells treated by camphorimine complexes **1**, **5**, and **9**. As shown in Figure 8, the ability of the complexes to activate caspase-3/7 in vitro could be correlated with their cytotoxicity. OVCAR3 cells treated with complex **1** exhibited low caspase activation, almost comparable with the controls (untreated cells). Cells treated with complexes **5** and **9** exhibited higher caspase-3 and -7 activation levels. Interestingly, complexes **5** and **9** were also found to induce high ROS levels in OVCAR3 cells.

#### 2.1.7. Complex Cellular Uptake

The cellular uptake is an important parameter to consider in the evaluation of the effects of metal-based complexes as therapeutic agents. To be effective, complexes must enter the cancer cells and reach their targets. The uptake of the specific metal complexes can be assessed quantitatively in bulk cell extracts using analytical techniques such as ICP-MS and PIXE. In addition, the micro-distribution of the relevant elements can be examined in single cells using the PIXE technique implemented in nuclear microscopy.

In this work, the metal intracellular concentrations were assessed by PIXE or ICP-MS in OVCAR3 cells treated with selected copper, silver, and gold complexes (**1**, **5**, and **9**). The selection was made taking into account, for each metal complex, its biological potential to be further studied as a therapeutic drug. Due to the high detection limit of PIXE for silver (300 ± 52 μg/g), the concentration of silver in the cells incubated with complex **5** was determined by ICP-MS. Table 3 summarizes the results obtained for cells treated with complexes **1** (copper), **5** (silver), and **9** (gold). Results clearly showed an expressive uptake of silver and gold by OVCAR3 cells, whereas a limited uptake of copper was registered taking into account the concentration of the complex in the cell medium.

As copper is a physiological trace element, untreated OVCAR3 cells were also included as a control sample for elemental analysis. The concentration of endogenous copper in OVCAR3 cells (12 ± 3.4 ng per million cells) was significantly lower than the concentration of copper determined in OVCAR3 cells treated with complex **1**.

The distribution of copper and gold in treated cells was inspected by nuclear microscopy, making use of the PIXE technique to map and assess elemental contents in single cells. The nuclear microscopy techniques offer several advantages, regarding relatively high spatial resolution and sensitivity for a wide range of elements with physiological roles and those with pharmaceutical interest, in whole cells, while preserving most of their in vivo conditions [24].

**Figure 9:**
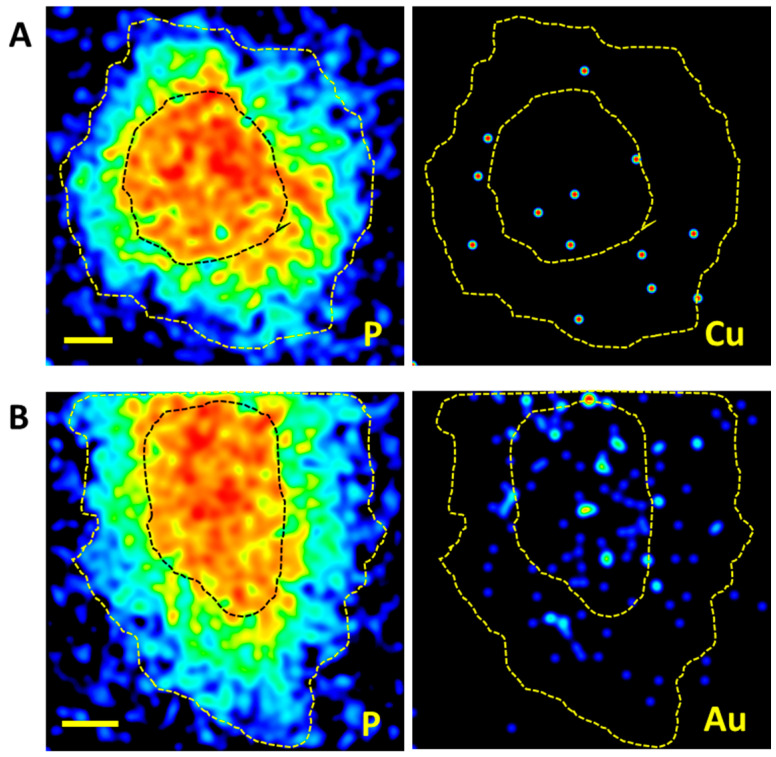
Nuclear microscopy images of OVCAR3 cells treated with complex **1** (**A**) and complex **9** (**B**). The cell morphology can be visualized in the map of phosphorus (P) distribution (cytoplasm (low content) and nucleus (high content)), to help interpreting the Cu and Au distributions. The gradient amount is represented by a dynamic color scale: low—blue to high—red. The dashed lines delineate the cell and nucleus contours. Scale bar: 5 μm.

Limited information could be obtained regarding the cellular distribution of Cu, due to its low concentration in cells. In contrast, the direct visualization of Au in cells treated with complex **9** clearly showed the uptake of Au and its profuse distribution across OVCAR3 cells, as illustrated in Figure 9. In fact, the modest uptake observed in extracts of cells treated with complex **1** is reflected in the uncharacteristic map of copper distribution across cells. In contrast, the gold uptake by cells treated with complex **9** suggests that the metal accumulated in the cell and spread in both the cytoplasm and the nucleus. The cellular localization of the metal is crucial to infer cellular targets [36]. However, further studies are needed to unravel possible targets. The reported results showed that cytotoxicity did not directly translate in higher uptake. The relevance of the correlation of metal uptake with the cytotoxic activity may be due to variations in the interaction of metal complexes with cellular components [37].

### 2.2. Toxicity Assessment of [Ag(NO_3_)(^1^A)] (5) Using the In Vivo Model C. elegans

The nematode *C. elegans* has been used as an alternative model to assess toxicity effects in mammal models. Although the experiments performed with these lower organisms need further validation, they are complementary to those performed using in vitro models, may have a good predictive power for effects in humans, and are part of the policy to reduce, refine, and replace animal testing (3R policy).

**Figure 10:**
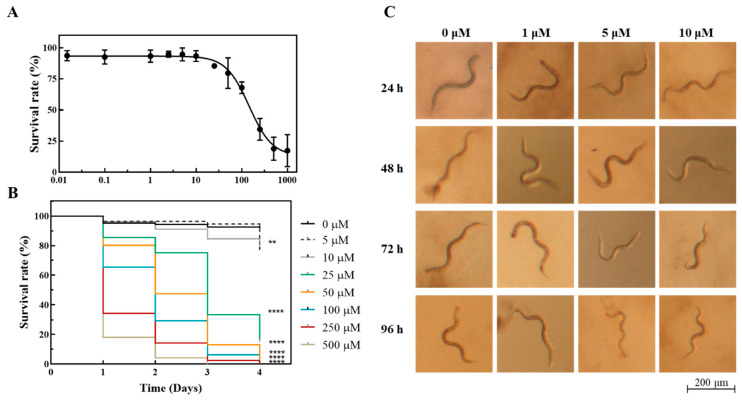
Toxicological evaluation of complex **5** using the *C. elegans* in vivo model. (**A**) Survival curve of *C. elegans* L4 larvae upon 24 h exposure to the indicated [Ag(NO_3_)(^1^A)_2_] concentrations. Error bars represent the standard error of the mean. (**B**) Survival curves of *C. elegans* treated with single concentrations of the complex and untreated control (no complex, 1% DMSO). Differences compared to the control were considered as significant with ** *p* < 0.001 and **** *p* < 0.0001 and were determined using the log-rank (Mantel–Cox) test. (**C**) Representative images of the *C. elegans* worms after 24 h, 48 h, 72 h, and 96 h exposure to the indicated concentrations of the complex. Scale bars: 200 μm.

The toxicity of the complex **5** toward *C. elegans* was screened following the survival of synchronized L4 nematodes after exposure to different concentrations of the complex every 24 h for a total of 96 h (4 days). After 24 h of incubation in the presence of the complexes, the lethal concentration (LC_50_) for [Ag(NO_3_)(^1^A)_2_] was estimated as 144.7 ± 1.2 µM (Figure 10A). The visual inspection of the cultures after 24 h of incubation revealed that the worms swam in a coordinated and fast manner for concentrations of complex **5** below 100 µM (Figure 10C and Figure S2). For higher concentrations of the compound, the movements of the surviving worms were sluggish and uncoordinated, while some worms were even paralyzed, presenting a distinct body posture (Supplementary Figure S2). Increasing the exposure time decreased the LC_50_. The LC_50_ values at 48 h, 72 h, and 96 h were, respectively, 55.18 ± 1.13 µM, 20.74 ± 1.06 µM, and 16.12 ± 1.057 µM. Upon 72 h of exposure to Ag(NO_3_)(^1^A)_2_ concentrations equal to or less than 10 µM, the survival rate, body size, and movement of nematodes were similar to the nematodes in the control (1% DMSO) (Figure 10B,C). The reproductive capacity of *C. elegans* exposed to [Ag(NO_3_)(^1^A)_2_] from the last larval stage (L4) to adulthood was also evaluated using different concentrations of the complex. The results show that the nematodes were able to progress into adulthood and produce eggs when exposed to concentrations of Ag(NO_3_)(^1^A)_2_ not higher than 5 µM.

## 3. Discussion

The current research on metal-based complexes is expected to contribute to the development of novel drugs, to increase the arsenal of available chemotherapeutics. Platinum(II) drugs like cisplatin and its analogues have been widely used in the clinic to fight against several types of cancers, acting by specifically targeting the DNA. A great percentage of patients undergoing chemotherapy receive a platinum drug, either alone or in combination with another chemotherapeutic drug. Despite their important role in cancer treatment, platinum drugs display systemic dose-dependent toxicity and, frequently, drug resistance, which leads to treatment failure. These limitations have strongly motivated the interest in the search for novel metal-based drugs as alternatives to cisplatin. Several metal complexes other than Pt-based have been developed and evaluated as prospective antitumor agents. For a great number of complexes, the mechanisms of action often rely on DNA-independent processes such as targeting specific cellular proteins and/or disruption of cellular signaling pathways often accompanied by oxidative stress. In addition to their ability to cause damage to lipids, proteins, and DNA, ROS are often used by eukaryotic cells as signaling molecules.

Cisplatin has been the most used drug for the treatment of ovarian cancer. These types of tumors at a later stage have a very poor prognosis and frequently acquire resistance to cisplatin. In the search for metal-based anticancer agents as alternative to cisplatin, earlier studies by Carvalho et al. showed that silver complexes with camphor ligands can exhibit interesting anticancer activities against both cisplatin-sensitive (A2780) and cisplatin-resistant (A2780cisR) ovarian cancer cells [22,23].

The main goal of the present work was the evaluation of the potential value of selected complexes of Cu(I) (**1**, **3**)-, Cu(II) (**2**, **4**)-, Ag(I) (**5**, **6**, **7**)-, and Au(I) (**8**, **9**)-containing camphor derivatives (camphorimines) for ovarian cancer treatment.

Results demonstrated a different cytotoxic profile for the three set of complexes. As the ligands did not induce significant cytotoxicity, it is possible to assume that the differences in cytotoxicity were not directly related to the ligands but related to the metal precursors used, particularly the silver and gold salts. The complexes presented herein highlight the importance of the metal site and the type of ligands for exploring metal-based complexes with potential biological applications.

When compared to the silver and gold camphorimine complexes, copper complexes present considerably lower cytotoxic activity, with IC_50_ values in the range 40–70 µM for both ovarian cancer cell lines. This observation could be the result of poor stability of the copper complexes in cell culture media, together with their low cellular uptake, as confirmed by UV/Vis spectra and PIXE, respectively. No appreciable differences in activity were observed for Cu(I) and Cu(II) complexes with the monocamphor (^1^A, ^2^A) and bicamphor (^1^B, ^2^B) ligands (complexes **1**–**3**), suggesting that the copper oxidation state is not a factor contributing to the complex cytotoxicity. In addition, the lower activity of these copper complexes is consistent with unchanged concentrations of cellular endogenous elements (e.g., P, K, Ca, and Zn), both in individual cells and in cell pellets.

Gold complexes also showed no appreciable differences in activity whether the complexation was with the monocamphor ^1^A or ^2^A ligands (**8**, **9**). However, in contrast with copper complexes, cellular uptake of gold was significantly higher, and the complexes exhibited higher cytotoxicity.

The silver nitrate (AgNO_3_) and silver acetate (Ag(CH_3_COO)) derived complexes showed distinct patterns of activity, attributed to the characteristics of the metal core. Silver acetate is slightly less cytotoxic than silver nitrate, and the derived complexes followed the same trend (**7** vs. **5**).

The high activity of the binuclear complex **6** with two silver atoms per molecule strengthens the relevance of the metal in the process.

Despite the relevant activity of the silver and gold complexes, the metal ion homeostasis in OVCAR3 cells seemed to be relatively preserved, since concentrations of most physiological elements were unchanged, with the exception of potassium for complexes **5** and **9**. This change in potassium concentrations may be a consequence of the increased lipoperoxidation and membrane permeability caused by ROS.

The mechanisms of action of these complexes cannot be attributed to the interaction with the DNA, as observed by electrophoresis using single-stranded DNA. None of the complexes led to changes in the electrophoretic mobility of ϕX174 DNA. We, therefore, conclude that none of the complexes interact significantly with the DNA molecule, in contrast to what has been found for cisplatin.

A great number of anticancer drugs in clinical use act by inducing oxidative stress in cancer cells and consequently cell death. The generation of ROS by the copper, silver, and gold complexes in the OVCAR3 cells (mainly peroxides and superoxide) was evaluated using a fluorescent probe and the tetrazolium salt (NBT) for the investigation of the oxidative metabolism in cells. The results obtained show that the silver and gold complexes induced the generation of ROS in a dose-dependent manner. Among the copper complexes, only complex **1** followed a similar trend.

ROS can react with membrane lipids to generate lipid peroxides that are further degraded into malondialdehyde (MDA) and 4-hydroxy-2-noneal (4-HNE). These end-products of lipid peroxidation have a cytotoxic role by promoting cell death.

After treatment of OVCAR3 cells with complexes **1**, **5**, and **9**, the MDA content was low but higher than the basal level of lipid peroxides in the untreated OVCAR3 cells. The MDA levels correlated with the ROS levels, suggesting that the mechanism of action of complexes **1**, **5**, and **9** involves, at least partially, the generation of ROS.

In addition to cultured cancer and normal cells, we used the nematode *C. elegans* to evaluate the toxicity of the most promising complex **5** in a living organism. Results showed that the LC_50_ at 24 h was 144.7 µM, well above the 2.24 µM determined for the OVCAR3 cells. These results encourage the continuation of our studies on higher organisms. Studies continue to explore the mechanism of action, particularly for complex **5** that revealed a promising biological profile and excellent selectivity, with cytotoxicity toward ovarian cancer cells while sparing normal cells.

To sum up, this study showed that metal camphorimine complexes could be prospective anticancer drugs with a better profile than cisplatin. Additionally, the study provided evidence for the biological properties of complexes being tuned by the metal, with the characteristics of the camphorimine ligands having a nonappreciable effect on the activity.

## 4. Materials and Methods

The complexes were synthesized under nitrogen using Schlenk and vacuum techniques. Complexes [{CuCl}_2_{OC_10_H_14_N(C_6_H_4_NH_2_)}] (**1**) [38], [{CuCl}_3_{(OC_10_H_14_N)_2_,μ-C_6_H_4_-*p*)}] (**3**) [39], [Ag(NO_3_)(OC_10_H_14_NC_6_H_5_)_2_] (**5**) [28], [{Ag(OC_10_H_14_NC_6_H_5_)}_2_(μ-O)] (**6**) [40], [Ag(OH)(OC_10_H_14_NC_6_H_4_NH_2_)]·CH_3_COOH (**7**) [41], and K[Au(CN)_2_(OC_10_H_14_NC_6_H_4_NH_2_)] [42] (**9**), and camphor ligands (OC_10_H_14_NY: Y = C_6_H_5_, C_6_H_4_NH_2_-*p* and (OC_10_H_14_N)_2_C_6_H_4_-*p*) [43] were prepared according to reported procedures. Gold potassium dicyanide, silver nitrate, silver acetate, copper chloride, camphor, and amines were purchased from Sigma Aldrich-Merck (Darmstadt, Germany). Acetonitrile (PA-grade) was purchased from Carlo Erba (Val-de-Reuil, France), purified by conventional techniques [44] and distilled before use. The FTIR spectra were obtained from KBr pellets using a JASCO FT/IR 4100 spectrometer. The NMR spectra (^1^H, ^13^C, DEPT, HSQC, HMBC) were obtained from DMSO solutions using Bruker Avance II+ (300 or 400 MHz) spectrometers. The NMR chemical shifts are referred to TMS (δ = 0 ppm). The redox properties were studied by cyclic voltammetry using a three-compartment cell equipped with Pt wire working and secondary electrodes and interfaced with a VoltaLab PST050 equipment. The cyclic voltammograms were obtained from NBu_4_BF_4_ solutions in CH_3_CN (0.10 M) as electrolyte. The potentials were measured in volts (±10 mV) versus SCE at 200 mV/s using [Fe(η^5^-C_5_H_5_)_2_]^0/+^ (*E* = 0.382 V; CH_3_CN) as the internal reference. The window of potential was established using a solution of the freshly prepared electrolyte.

### 4.1. Synthesis

[CuCl_2_((OC_10_H_14_N)_2_C_6_H_4_-*p*)] (**2**): The bicamphor ligand (OC_10_H_14_N)_2_C_6_H_4_-*p* (350 mg, 0.87 mmol) was added to a THF solution (5 mL) of CuCl_2_·2H_2_O (133 mg, 0.78 mmol) and the mixture was stirred at room temperature (RT) for 4 h. The formed brownish precipitate was filtered off and dried. Yield 85%. FTIR (KBr, cm^−1^): 1751, 1690. Elemental analysis (%) for CuCl_2_C_26_H_32_N_2_O_2_: found: C, 57.8; N, 5.1; H, 5.9; calculated: C, 57.9; N, 5.2; H, 5.9.

[CuCl_2_(OC_10_H_14_NC_6_H_5_)]·HCl·½H_2_O (**4**): CuCl_2_·2H_2_O (33 mg, 0.78 mmol) and OC_10_H_14_NC_6_H_5_ (210 mg, 0.87 mmol) were dissolved in THF (5 mL), and the mixture was stirred at RT for 4 h. Upon filtration to remove the light suspension, the compound was obtained as a green precipitate upon partial solvent evaporation and filtration. Yield 53%. FTIR (KBr, cm^−1^): 3434 (H_2_O), 1742 (CO), 1653 (CN). Elemental analysis (%) for CuCl_2_C_16_H_19_NO·HCl½·H_2_O: found: C, 45.2; N, 3.2; H, 4.8; calculated: C, 45.6; N, 3.3; H, 5.0.

K_2_[{Au(CN)_2_}_2_(OC_10_H_14_NC_6_H_5_)_3_] (**8**): KAu(CN)_2_ (50 mg, 0.17 mmol) and ligand ^1^A (OC_10_H_14_NC_6_H_5_; 82 mg, 0.34 mmol) were stirred under vacuum for 0.5 h. Acetonitrile (7 mL) was added, forming a yellow solution. The reaction was stirred at RT overnight. The formed light suspension was filtered off and discarded. The solvent was pumped to dryness affording the compound as a yellow precipitate. Yield 72%. FTIR (KBr, cm^−1^): 2141 (CN), 1743 (CO), 1663 (CN), 1652 (CH_Ph_). Elemental analysis (%) for K_2_Au_2_C_52_H_57_N_7_O_3_: found: C, 48.3; N, 7.5; H, 4.4; calculated: C, 48.0; N, 7.5; H, 4.4. ^1^H-NMR (DMSO, 300 MHz, δ_ppm_): 7.40 (d, *J* = 7.7 Hz, 2H), 7.18 (t, *J* = 7.4 Hz, 1H), 6.89 (d, *J* = 7.9 Hz, 2H), 2.70 (d, *J* = 4.6 Hz, 1H), 2.11–2.00 (m, 1H), 1.93–1.80 (m, 1H), 1.67–1.45 (m, 2H), 0.98 (s, 3H), 0.93 (s, 3H), 0.79 (s, 3H). ^13^C-NMR (DMSO, 300 MHz, δ_ppm_): 205.7 (C2), 171.7 (C3), 149.5 (C(≡N)_Au_; C_Ph-ipso_); 129.2 (C_Ph_), 125.1 (C_Ph_), 119.9 (C_Ph_), 57.6 (C1), 49.6 (C4), 44.1 (C7), 29.5 (C6), 23.6 (C5), 20.4, 17.1 (C9, C10), 9.0 (C8).

### 4.2. Biological Assays

#### 4.2.1. Complex Cytotoxicity Activity

The complex cytotoxic activity was evaluated in human ovarian cancer cells A2780 (Sigma-Aldrich, Darmstadt, Germany) and OVCAR3 (ATCC), human dermal fibroblasts (HDF, Sigma-Aldrich), and hamster lung fibroblasts V79 (ATCC). Cells were grown in RPMI 1640 medium (ovarian and V79 cells) supplemented with 10% FBS or fibroblast growth medium (HDF) and incubated at 37 °C, 5% CO_2_, in a humidified atmosphere (Heraeus, Hanau, Germany). Cell viability was assessed by the MTT assay [45,46]. In a typical experiment, cells were seeded in 96-well plates at a density of 10^4^ cells/well in 200 μL medium and allowed to adhere overnight. The medium was then discarded, and 200 µL of fresh medium containing each compound in the concentration range 10^−7^–10^−4^ M was added to the cells for 24 h treatment. Complexes were first dissolved in DMSO and then in medium to prepare the serial dilutions. After 24 h incubation, the solutions were aspirated, and 200 µL of MTT solution (0.5 mg/ mL) was added to each well. After 3 h, at 37 °C, the MTT solution was aspirated, and the formazan crystals formed were solubilized with DMSO. The absorbance at 570 nm was measured using a plate spectrophotometer (Power Wave Xs, Bio-Tek, Santa Clara, CA, USA). The IC_50_ values were calculated from dose–response curves using the GraphPad Prism software (vs. 5.0).

#### 4.2.2. Detection of ROS by H_2_DCF-DA

Intracellular ROS levels, mainly H_2_O_2_, were measured using dihydro-2′7′dichlorofluorescein diacetate (H_2_DCF-DA) [45,47]. This cell-permeable molecular probe is de-esterified within the cell and turns to highly fluorescent 2′,7′-dichlorofluorescin (DCF) upon oxidation with ROS. For the assays, OVCAR3 cells (2 × 10^4^ cells /well) were seeded in 96-well plates and left to adhere overnight. The medium was then replaced with a solution of 10 μM H_2_DCF-DA in colorless DMEM (FluoroBrite™ DMEM, Gibco^®^, Waltham, MA, USA) and cells were incubated at 37 °C for 30 min. Then, the medium was aspirated, and cells were incubated with the compounds in fresh medium at selected concentrations for 1 h. DCF fluorescence was measured using a Varioskan Lux multimode microplate reader (ThermoFisher Scientific, Waltham, MA, USA) at 492 nm excitation and 517 nm emission. Results of fluorescence were expressed as the fold change in fluorescence levels compared with controls.

#### 4.2.3. Generation of Superoxide Radicals (NBT Assay)

The nitro blue tetrazolium (NBT) assay was carried out adapting a previously described method [35]. This assay was used to determine the ability of cells to produce superoxide anion upon treatment with the compounds. During the treatment, NBT is reduced by superoxide anion, resulting in dark-blue formazan particles. Briefly, after incubation of OVCAR3 cells with the camphorimine ligands and corresponding complexes in medium at selected concentrations for 1 h at 37 °C, 20 μL of a 10 mg/mL NBT solution in water was added to the cell’s medium, and incubation was subsequently prolonged for 1 h at 37 °C. Then, the medium was discarded, and the blue formazan particles were dissolved in 200 μL of 90% DMSO (90% DMSO:10% NaOH 0.1 N with 0.1% SDS). The resulting NBT formazan was measured at 560 nm using a plate spectrophotometer.

#### 4.2.4. Lipid Peroxidation (MDA) Assay

The malondialdehyde (MDA) content in lipid extracts of OVCAR3 cells was measured using a colorimetric kit (BioVision, Milpitas, CA, USA), a method that relies on the reaction with thiobarbituric acid (TBARS assay) [48]. Briefly, OVCAR3 cells (5 × 10^5^ cells/2 mL medium) were seeded in six-well plates and left to adhere for 24 h. Then, after treatment with the complexes at selected concentrations for 24 h at 37 °C, the medium was removed, the cells were washed with PBS, trypsinized, and centrifuged. The resulting cell pellet was treated following the kit recommended protocol. In brief, the cells were lysed and centrifuged, and then 200 μL of the supernatant was added to 600 μL of thiobarbituric acid (TBA) to generate the red-pink MDA–TBA adduct (TBARS) incubating the mixture for 1 h at 95 °C. Then, 200 μL aliquots were then taken and transferred to a 96-well plate for MDA quantification at 532 nm. An MDA calibration curve with MDA standards was made in parallel with the samples, using the same procedure.

#### 4.2.5. Complex Interaction with DNA

The DNA binding activity of the metal complexes was assessed through their ability to alter the electrophoretic mobilities of the covalently closed circular (ccc) and open circular (oc) forms of ΦX174 supercoiled DNA as previously described [30]. Briefly, a mixture containing 200 ng of ϕX174 DNA (Promega, Madison, WI, USA) and different concentrations of the metal complexes was prepared. After incubation for 24 h at 37 °C in the dark, the samples were run in a 0.8% agarose gel in TAE buffer for 3 h at 90 V. The gel obtained was then stained using a 3× GelRed^®^ (Biotium, Fremont, CA, USA) solution in H_2_O and imaged in an AlphaImagerEP (Alpha Innotech, San Leandro, CA, USA).

#### 4.2.6. Apoptosis (Caspase-3/7 Assay)

The activities of caspase-3 and -7 were assessed using the Caspase-Glo^®^ 3/7 assay (Promega, Madison, WI, USA). The assay kit provides a proluminescent caspase-3/7 substrate (DEVD-aminoluciferin) and luciferase in a reagent optimized for caspase-3/7 activity, luciferase activity, and cell lysis. In the presence of caspase 3/7, this substrate is cleaved, and aminoluciferin is released and consumed by luciferase, which generates a luminescent signal proportional to the caspase activity present in the cells. The assay was carried out in a 96-well plate format with the OVCAR3 cells treated with complexes **1**, **5**, and **9** for 24 h at concentrations below their IC_50_ according to a previously described method [46]. After 24 h of incubation with the complexes, 100 μL of medium was removed from each well. Caspase 3/7^®^ reagent was added in a 1:1 ratio, and the plate was shaken in an orbital shaker for 30 s at 300−500 rpm. The plate was them incubated at RT for 1.5 h, protected from light. Dichlorofluorescein (DCF) fluorescence was measured at 492 nm excitation and 517 nm emission wavelengths, using a Varioskan LUX scanning multimode reader (Thermo Fisher Scientific, Waltham, MA, USA). Results (mean ± SD) were expressed as relative luminescent units (RLU).

#### 4.2.7. Complexes Cellular Uptake

The intracellular concentrations of copper, silver, and gold were determined using the particle-induced X-ray emission (PIXE) technique, installed at the Van de Graaf accelerator of Instituto Superior Técnico. For this purpose, OVCAR3 cells were incubated with complexes **1** (24 h) and **9** (6 h), at concentrations corresponding to the IC_50_, i.e., 70 μM for complex **1** and 20 μM for complex **9**. The cell pellets obtained by centrifugation after washing the cells with PBS to remove the culture medium were freeze-dried and subjected to microwave-assisted acid digestion. The detailed methodology encompassing sample treatment, PIXE analysis, and concentration calculations was previously described [49]. The elemental concentrations were obtained in µg/g (of dry or wet material) and converted to µg/10^6^ cells. The minimum detection limits on a dry weight basis for Cu and Au were 7.2 ± 0.5μg/g and 47 ± 2 μg/g, respectively.

#### 4.2.8. Imaging Copper and Gold Distribution in Cells

OVCAR3 cells (ca. 1 × 10^6^) were prepared for nuclear microscopy experiments by seeding the cells on a 100 nm thick silicon nitride membrane (Silson Ltd., Southam, UK), contained in six-well plates, and incubated overnight as described previously [27]. Cells were incubated with complexes **1** and **9** at concentrations corresponding to the IC_50_ (45 μM for 24 h and 30 μM for 3 h, respectively). After incubation, the cells were analyzed at the nuclear microscopy facility of the C2TN/IST with a 2.0 MeV proton beam focused down to 3 × 4 µm^2^ dimensions [50]. Images of the micro-distributions of Cu and Au in OVCAR3 cells, as well as minor and trace physiological elements (e.g., P, S, K, and Fe), were obtained using the PIXE multielemental technique. Images of whole OVCAR3 cells were taken with 30 × 30 µm^2^ scan sizes. Acquisition of data imaging and spectral analysis was performed using OMDAQ 2007 (Oxford Microbeams Ltd., Oxford, UK) [51].

#### 4.2.9. In Vivo Studies Using the Nematode *Caenorhabditis elegans*

The *C. elegans* Bristol strain N2 was used in the present work. The nematode strain, obtained from the Caenorhabditis Genetic Center, was maintained at 20 °C on Nematode Growth Medium (NGM) agar plates seeded with *Escherichia coli* OP50 under standard conditions and synchronized using standard protocols and as previously described [52,53,54]. L4 synchronized worms, prepared as previously described [53,54], were exposed to [Ag(NO_3_)(^1^A)_2_] (**5**) complex final concentrations of 0, 1, 2.5, 5, 10, 25, 50, 100, 250, and 500 µM in M9 buffer (22.04 mM KH_2_PO_4_, 42.27 mM Na_2_HPO_4_, 85.56 mM NaCl, and 1 mM MgSO_4_) supplemented with 0.2% (*w*/*v*) of dead *E. coli* OP50, for 96 h at 20 °C, in a final volume of 500 μL (24 wells) [55]. The total number of living and dead worms per well was assessed at 24, 48, 72, and 96 h timepoints with the aid of a Zeiss Stemi 2000-C stereomicroscope. The survival of live worms was computed by tapping the plate and counting the moving worms. Three independent experiments were performed, and each well contained 15 ± 5 L4 worms (total number of worms *n* = 1543). Concentration–response curves and Kaplan–Meier curves were drawn using GraphPad Prism (GraphPad Software, San Diego, CA, USA). Concentrations that resulted in the death of 50% of the tested organisms (LC_50_) were determined by fitting the concentration response curve to a four-parameter dose–response curve. Worm images were captured using an AxioCam 503 color device coupled to the Zeiss Stemi 2000-C Stereo Microscope.

## 5. Conclusions

The cytotoxic activities of nine camphorimine complexes [Cu(I) (**1**, **3**), Cu(II) (**2**, **4**) Ag(I) (**5**, **6**, **7**), and Au(I) (**8**, **9**)] and their precursors [CuCl, CuCl_2_, Ag(NO_3_), and Ag(CH_3_COO)] against A2780 and OVCAR3 ovarian cancer cells were evaluated showing that the Ag and Au complexes and their metal precursors are highly active, while the Cu complexes and their precursors presented a moderate to low activity. The characteristics of the camphorimine ligands, either of the mono- or bicamphor type, displayed just a slight effect on the cytotoxicity of the complexes, which was attributed to the lipophilic character of the camphorimine substituent (^1^A: 2.94 ± 0.58; ^2^A: C_6_H_4_NH_2_, 2.25 ± 0.59). The free camphorimine ligands did not induce appreciable cytotoxicity (IC_50_ > 100 µM).

The stability of the complexes in cellular media was evaluated by UV/Vis spectroscopy, showing that the silver and gold complexes were stable in the cell medium, maintaining their original form. In contrast, the copper complexes displayed instability in the absence of FBS the medium serum supplement.

The anticancer activity of the complexes followed the order Au(I) > Ag(I) > Cu(I) ≈ Cu(II), with the silver complexes (**5**–**7**) showing the highest selectivity toward the ovarian cancer cells, as confirmed by the selectivity indices.

The cytotoxicity of complexes **1**, **5**, and **9** correlated well with their cellular uptake and stability. In fact, the quantification by PIXE of the metal in the OVCAR3 cells indicated only vestigial concentrations of copper (**1**), while reasonable levels of silver (**5**) and gold (**9**) were observed.

The herein results point to the mechanism of cell death promoted by the ability of the camphorimine complexes under study to induce different types of oxygen radicals (ROS), namely, peroxides and superoxide, which enhanced the formation of lipoperoxides, as demonstrated for selected compounds (**1**, **5**, and **9**).

The cytotoxicity of the complexes toward the nontumoral HDF and V79 fibroblasts was evaluated showing that, in general, all the complexes were more cytotoxic toward the ovarian cell lines than toward the nontumoral cells. Compared to cisplatin, the silver camphorimine complexes had a better biological profile. To get further details on the toxicity of complex **5** that displayed the highest selectivity toward the OVCAR3 ovarian cancer cells, the in vivo model *C. elegans* was used, showing that the complex was nontoxic for concentrations (5 µM) well above the complex IC_50_ (2.24 ± 0.48 µM).

## Figures and Tables

**Table 1:** IC_50_ values ^a^ and reduction potentials for the camphorimine complexes.

Complexes		$E_{p}^{red}\;(\mathrm{Volt}){\;}^{b}$	A2780	OVCAR3	V79	HDF	SI ^c^
[{CuCl}_2_(^2^A)]	**1**	−0.49	45.6 ± 11	72.4 ± 9.1	34.5 ± 9.7	116 ± 27	1.6
[CuCl_2_(^1^B)]·2H_2_O	**2**	0.48 ^d^	49.5 ± 14	37.6 ± 8.5	>100	>100	>3
[(CuCl)_3_(^2^B)]	**3**	−0.46 ^d^	43.1 ± 9.1	37.5 ± 7.7	44.9 ± 10	48.3 ± 27	1.3
[CuCl_2_(^1^A)]·HCl·½H_2_O	**4**	0.47 ^d^	>100	115 ± 25	>100	>100	>1
CuCl		−0.46		>100			
CuCl_2_		0.58 ^d^		>100			
[Ag(NO_3_)(^1^A)_2_]	**5**	0.12	3.53 ± 0.90	2.24 ± 0.48	>100	>100	>50
[{Ag(^1^A)}_2_(μ-O)]	**6**	0.00	0.66 ± 0.28	0.63 ± 0.23	3.01 ± 0.9	30.6 ± 8.5	49
[Ag(OH)(^2^A)]CH_3_COOH	**7**	−0.047	10.4 ± 2.9	8.99 ± 3.3	34.1 ± 15	>100	31
Ag(NO_3_)		0.18		2.66 ± 1.0			
Ag(CH_3_COO)		−0.043		3.38 ± 2.0			
K_2_[{Au(CN)_2_}_2_(^1^A)_3_]·½H_2_O	**8**	−1.65	0.08 ± 0.01	0.08 ± 0.03	0.48 ± 0.06	0.46 ± 0.17	5.7
K[Au(CN)_2_(^2^A)]·H_2_O	**9**	−1.85	0.04 ± 0.02	0.07 ± 0.01	0.48 ± 0.30	0.59 ± 0.11	8.4
KAu(CN)_2_				0.49 ± 0.02			

^a^ Values in μM, determined through MTT method after 24 h incubation. ^b^ Potential measured in volts (±10 mV) using Bu_4_NBF_4_/CH_3_CN as electrolyte. ^c^ SI = $\mathrm{selectivity}\;\mathrm{index}=\frac{IC50\left(HDF\right)}{IC50\left(OVCAR3\right)}$ > 100, which indicates that, at 100 µM, the cellular viability (% control) was higher than 80% and could not be determined with the GraphPad prism software. ^d^ $E_{1/2}^{red}$ [28].

**Table 2:** Lipid peroxide (MDA) content in the OVCAR3 cells treated for 6 h with **1**, **5**, and **9** at 100, 50, and 20 μM, respectively. Results are the mean ± SD.

Complexes	MDA (pmoles/10^6^ Cells)
**1**	290 ± 15
**5**	1740 ± 270
**9**	1370 ± 210
Control	7 ± 4

**Table 3:** Total cellular uptake of OVCAR3 cells treated with complexes **1** (Cu), **5** (Ag), and **9** (Au) using the IC_50_ equivalent concentrations of 70 µM (**1**, 24 h), 20 µM (**5**, 6 h), and 20 µM (**9**, 6 h). The results are the mean ± SD values of concentrations expressed in ng (Cu, Ag, or Au)/million OVCAR3 cells.

Complex	Metal	Concentration (ng Metal Content/10^6^ Cells)
**1**	Cu	54 ± 4
**5**	Ag	273 ± 15
**9**	Au	688 ± 23

## Data Availability

Data is contained in this manuscript and supplementary material.

## Supplementary Materials

The following supporting information can be downloaded at https://www.mdpi.com/article/10.3390/antibiotics11081010/s1: Figure S1. Production of ROS (relative to untreated cells) in OVCAR3 cells; Figure S2. Toxicological evaluation in the *C. elegans* in vivo model.
