# Supplementary material for: Tuning the Biological Activity of Camphorimine Complexes through Metal Selection
Source: Antibiotics (Basel). 2022 Jul 27;11(8):1010. doi: 10.3390/antibiotics11081010 (PMC9405135; doi:10.3390/antibiotics11081010)
Supplement: Supplementary file 1 [file antibiotics-11-01010-s001.zip › antibiotics-1839502-supplementary.pdf]

SUPLEMENTARY INFORMATION

Tuning the biological activity of camphorimine complexes through metal selection

Joana P. Costa [1], Teresa Pinheiro [2], Maria S. Martins [3,4], M. Fernanda N. N. Carvalho [1,*], Joana R. Feliciano [5], Jorge H. Leitão [5], Rafaela A. L. Silva [3], Joana F. Guerreiro [3], Luís M. C. Alves [3], Inês Custódio [3,4], João Cruz [4] and Fernanda Marques [3,*]

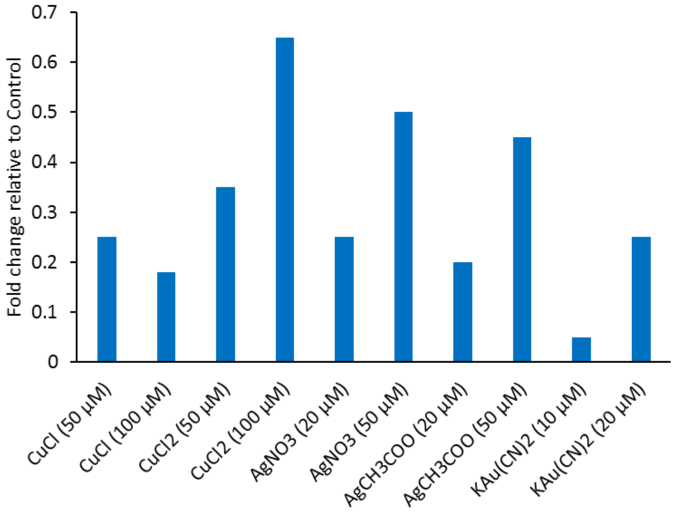

Figure S1. Production of ROS (relative to untreated cells) in OVCAR3 cells. Cells were treated with the metal precursors CuCl and $CuCl_2$ at 50 and 100 µM, $AgNO_3$ and $AgCH_3COO$ at 20 and 50 µM and $KAu(CN)_2$ at 10 and 20 µM using the H_2DCFDA method.

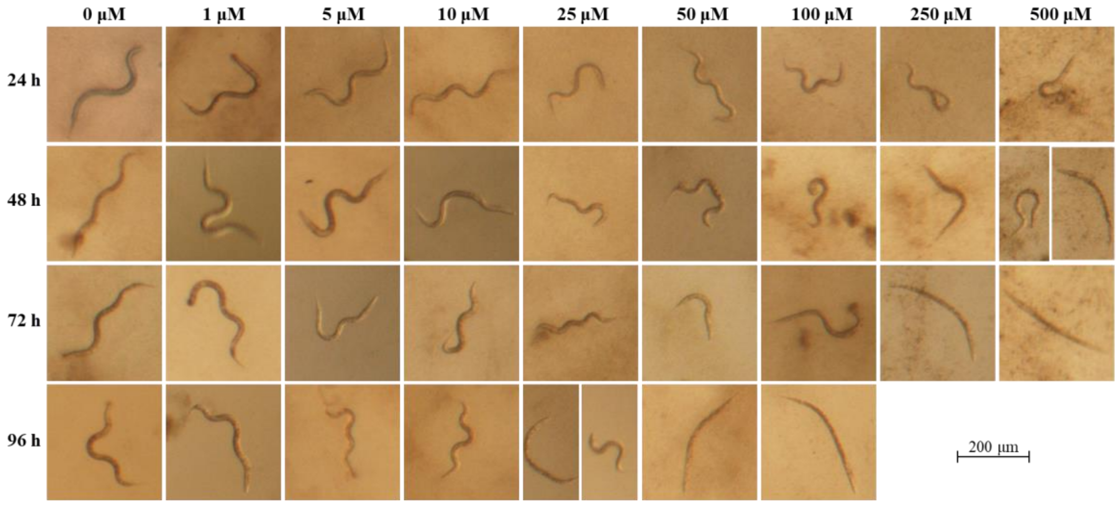

Figure S2. Toxicological evaluation of $[Ag(NO_3)(^1A)_2]$ (**5**) in the *C. elegans in vivo* model. Representative images of the *C. elegans* worms were captured after 24 h, 48 h, 72 h and 96 h of exposure to the indicated concentrations of the complex. Scale bars are 200 µm.
